# Grimace scale assessment during *Citrobacter rodentium* inflammation and colitis development in laboratory mice

**DOI:** 10.3389/fvets.2023.1173446

**Published:** 2023-06-05

**Authors:** Pia Pascale Peppermüller, Jonathan Gehring, Eva Zentrich, André Bleich, Christine Häger, Manuela Buettner

**Affiliations:** Institute for Laboratory Animal Science, Hannover Medical School, Hannover, Germany

**Keywords:** mouse grimace scale, infection, chronic inflammation, surgery, clinical score

## Abstract

**Introduction:**

Bacterial infections and chronic intestinal inflammations triggered by genetic susceptibility, environment or an imbalance in the intestinal microbiome are usually long-lasting and painful diseases in which the development and maintenance of these various intestinal inflammations is not yet fully understood, research is still needed. This still requires the use of animal models and is subject to the refinement principle of the 3Rs, to minimize suffering or pain perceived by the animals. With regard to this, the present study aimed at the recognition of pain using the mouse grimace scale (MGS) during chronic intestinal colitis due to dextran sodium sulfate (DSS) treatment or after infection with *Citrobacter rodentium*.

**Methods:**

In this study 56 animals were included which were divided into 2 experimental groups: 1. chronic intestinal inflammation (*n* = 9) and 2. acute intestinal inflammation (with (*n* = 23) and without (*n* = 24) *C. rodentium* infection). Before the induction of intestinal inflammation in one of the animal models, mice underwent an abdominal surgery and the live MGS from the cage side and a clinical score were assessed before (bsl) and after 2, 4, 6, 8, 24, and 48 hours.

**Results:**

The highest clinical score as well as the highest live MGS was detected 2 hours after surgery and almost no sign of pain or severity were detected after 24 and 48 hours. Eight weeks after abdominal surgery B6-*Il4/Il10-/-* mice were treated with DSS to trigger chronic intestinal colitis. During the acute phase as well as the chronic phase of the experiment, the live MGS and a clinical score were evaluated. The clinical score increased after DSS administration due to weight loss of the animals but no change of the live MGS was observed. In the second C57BL/6J mouse model, after infection with *C. rodentium* the clinical score increased but again, no increased score values in the live MGS was detectable.

**Discussion:**

In conclusion, the live MGS detected post-operative pain, but indicated no pain during DSS-induced colitis or *C. rodentium* infection. In contrast, clinical scoring and here especially the weight loss revealed a decreased wellbeing due to surgery and intestinal inflammation.

## Introduction

According to the legal requirements in animal experimentation, the severity of any procedure inflicted on animals needs to be classified as mild, moderate, or severe according to the respective intensity of the experienced pain, suffering, or distress (EU directive 2010/63) (1). During the experiment, researchers commonly use clinical score sheets to monitor the actual experienced severity of individual animals. To cover all facets of the experienced severity, including pain, suffering, and distress, the assessment should follow a multivariate concept in which clinical scoring is supplemented by behavioral tests (2).

The International Association for the Study of Pain defines pain as an “unpleasant sensory and emotional experience associated with, or resembling that associated with, actual or potential tissue damage” (3). Although pain is a multidimensional phenomenon, it can be broadly classified into neuropathic and nociceptive pain. The activation of nociceptors due to actual or threatened damage to non-neural tissue leads to nociceptive pain. Neuropathic pain on the other hand is caused by a lesion or disease of the somatosensory nervous system (4). Pain emerging from the internal organs is defined as visceral pain and is usually difficult to localize as compared to somatic pain. Quite often, visceral pain does not correlate with actual visceral trauma but can also be caused by other factors, such as genetic factors, psychological stress, and the nature of the predisposed disease (5). Chronic visceral pain is often observed as a result of functional bowel disorders with patients complaining of symptoms such as cramping, abdominal pain, bloating, constipation, and/or diarrhea (6). There are several animal models, including inflammatory models, to study the implications of chronic visceral pain.

Ulcerative colitis and Crohn's disease are widespread inflammatory bowel diseases (IBD) with an incidence range of 12–26 per 100,000 people in the Western world (7). Due to the chronicity and relapsing nature of the disease, IBD significantly impairs the quality of life and performance of affected individuals (7, 8). The pathological mechanisms are still unclear, because of the complex interplay of microbial, environmental, and genetic factors leading to a disrupted intestinal barrier (9). To unravel the complex pathogenesis of IBD, animal models are still necessary for preclinical research.

Most frequently used are mouse models for IBD including the well-studied genetically engineered interleukin-10-deficient mouse (*Il10*^−/−^) (10). Histopathological hallmarks of *Il10*^−/−^ mice are inflammatory cell infiltration of the lamina propria and submucosa, epithelial hyperplasia, mucin depletion, crypt abscesses, ulceration, and thickening of the intestinal wall (11). Colitis development in *Il10*^−/−^ mice starts spontaneously after weaning and is microbiota dependent (9, 12). During experimentation with these mice, the administration of dextran sodium sulfate (DSS) or bacterial infection (e.g., *C. rod*.) is used to trigger intestinal inflammation. DSS is a water-soluble sulfated polysaccharide that incites inflammation by disrupting the epithelial monolayer that destroys interepithelial cell tight junctions and reduces mucin levels while simultaneously altering the resident microbiota (13). A dysbalanced microbiota and an impaired barrier integrity result in an inflammatory response (14). Other triggers can be a *C. rod*. infection, which causes weight loss and diarrhea but also colonic crypt hyperplasia, immune cell infiltration, and goblet cell depletion (15, 16). However, in terms of the refinement principle by Russell and Burch, in such animal models, the minimization of experienced pain, suffering, or distress requires as a first step, a precise severity assessment (17).

The burdens of intestinal inflammation in mice are without question of multidimensional quality, and like in humans, the main symptoms are diarrhea and pain. To monitor the disease development, researchers usually apply model-specific clinical score sheets, which evaluate general health status, body weight, and stool consistency (18), however, pain-specific indicators are usually not included. To obtain a more precise picture, variables indicating pain, suffering, and distress need to be implemented for severity assessment in animal experiments where inflammation-induced pain can occur. For the assessment of pain, several methods are available. Among the algesiometry assays, e.g., the von Frey test, there are also behavioral tests available such as the burrowing behavior or voluntary wheel running [for review (19)].

The scoring of facial expressions has been shown as a good parameter for the detection of postoperative pain in mice, and the so-called mouse grimace scale (MGS) is a frequently applied method in laboratory animal science (20). The MGS was established and widely used to assess pain in mice after laparotomy (21). The MGS scores the five facial action units, namely, orbital tightening, nose bulge, cheek bulge, ear position, and whisker change, and higher scores are indicative of pain (20). Grimace scales are also available for other species (22–25), and the development of automated tools became a milestone (26, 27). Unfortunately, the MGS is still a retrospective assessment and refinement possibilities are limited, so it has been adjusted as a cage-side observation method. In a study comparing grimace scaling based on video footage and cage-side observation, the authors found sex- and strain-independent lower live scores in untreated mice. In contrast, in a mouse model of colorectal cancer, the authors found no differences between the performance of video and live MGS (28–30). However, the detected scores were only slightly elevated in all treatment groups of this study (28). In a study comparing video vs. cage-side rat grimace scaling, the authors showed real-time grimace scoring as an indicator of pain in rats (31).

As the applicability of cage-side MGS for pain assessment is still largely unexplored, the aim of this study was the evaluation of the live mouse grimace scale (lMGS) as an indicator of pain during intestinal inflammation in mice. In a mouse model for chronic intestinal inflammation in B6.Cg-^Il4*tm*1*NntIl10tm*1*Cgn*^ (B6-*Il4*/*Il*10^−/–^) mice triggered by DSS or *C. rod.*, infection of the lMGS was assessed, and the results were compared to postoperative lMGS of the same animals.

## Materials and methods

### Mice

For this study, 47 female C57BL/6J (B6J) and ninde male and female B6.Cg-^Il4*tm*1*NntIl10tm*1*Cgn*^ (B6-*Il4*/*Il*10^−/–^) were bred at the Central Animal Facility of the Hannover Medical School and were used at the age of 8–10 weeks. B6-*Il*10^−/–^ mice were housed in filter-top cages located in a room with a controlled environment (21 ± 2°C, 60 ± 10% relative humidity, 12–14 air changes per hour) and 12-h light/dark cycle. If not stated otherwise, the mice received a pelleted diet (Altromin 1324 TPF, Altromin Spezialfutter GmbH & Co. KG, Lage, Germany) and autoclaved water *ad libitum*. Cages were lined with softwood granulate (poplar wood, ANT-Tierhaltungsbedarf, Buxtehude, Germany). The mice received Sizzle Nest paper material, a cotton nesting pad, and a mouse house (all ANT-Tierhaltungsbedarf). Animals were monitored according to FELASA recommendations (32) and did not reveal any evidence of infection with common murine pathogens except for *Helicobacter* sp., *Klebsiella oxytoca, Rodentibacter* sp, *Staphylococcus aureus, Chilomastix* sp., and *Trichomonas* sp. Healthy animals were included, whereas animals that already had intestinal inflammation before the start of the experiment were excluded from the study.

### Ethics statement

This study was conducted in accordance with German animal protection law and with the European Directive 2010/63/EU. All experiments were approved by the Local Institutional Animal Care and Research Advisory Committee (Hannover Medical School) and permitted by the Lower Saxony State Office for Consumer Protection and Food Safety (LAVES; file number: 20/3445).

### Study design

#### Experimental group 1: chronic intestinal inflammation

**Figure 1:**
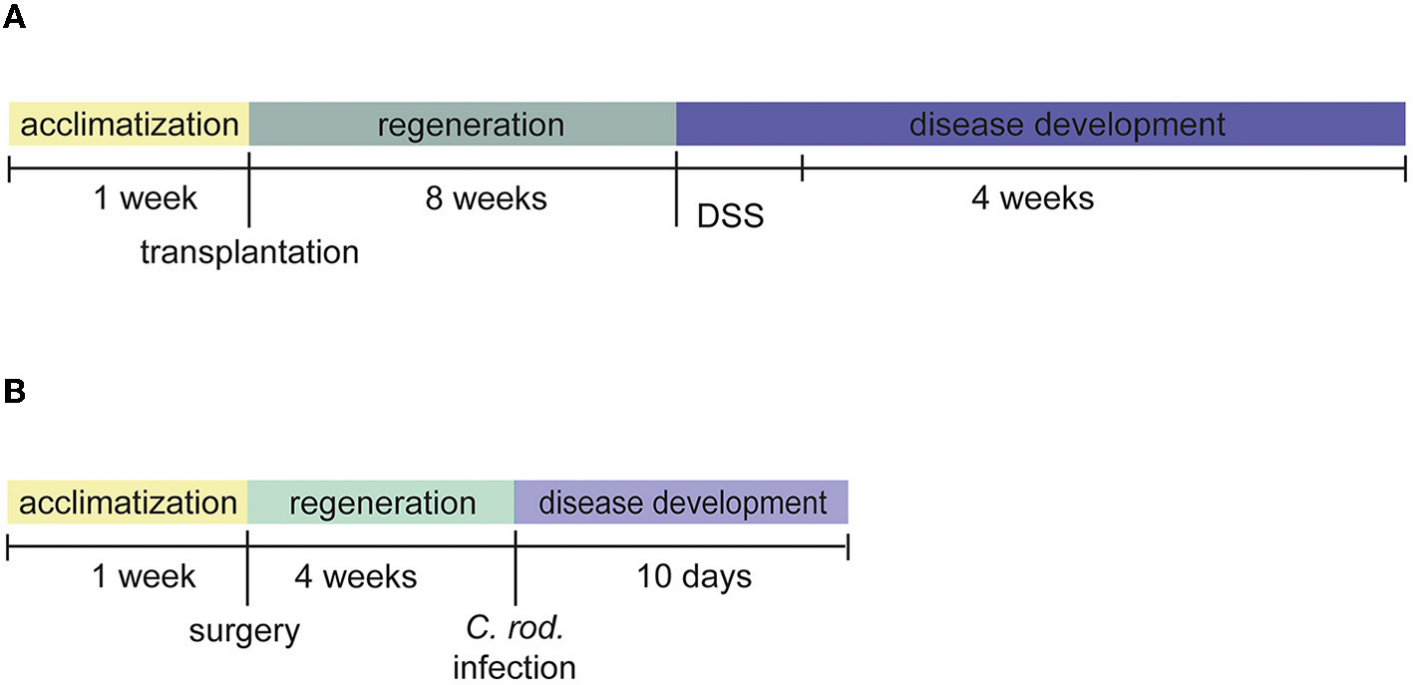
Experimental setup of the mouse models. **(A)** Chronic colitis of B6-*Il4*/*Il*10^−/−^ was induced 8 weeks after lymph node transplantation using DSS. **(B)** Acute colitis was induced 4 weeks after abdominal surgery by *C. rod*.

After surgical intervention of changing original mesenteric lymph nodes (mLN), female and male B6-*Il4*/*Il*10^−/−^ mice were divided into two experimental groups. The first group (*n* = 4) got transplanted donor mLN from another B6-*Il4*/*Il*10^−/−^ mouse (mLNtx) as a control group. The second group (*n* = 5) got transplanted mLNtx and received 2% DSS (Figure 1A).

#### Experimental group 2: acute intestinal inflammation

Female B6J animals were divided into two experimental groups: sham surgery with (*n* = 23) and without (*n* = 24) *C. rodentium* infection (Figure 1B).

All mice were group housed. Fifty-six animals were included in the experimental study; however, one mouse reached clinical score termination criteria because of colitis induction. All animals were sacrificed by CO2 inhalation (1.5 L/min gradient filling rate) followed by cervical dislocation. Allocation into the respective 2% DSS treatment or *C. rod*. infection groups was randomized. Clinical scoring and lMGS assessment were not performed blinded for the treatment groups. Analysis of intestinal histology was evaluated blinded.

#### Intestinal surgery

Sham operations were performed for B6J, and in the case of B6-*Il*4/*Il*10^−*/*−^ mice, donor LNtx was transplanted into the mesentery of the intestine. Mice were anesthetized with combined anesthesia of ketamine (Anesketin^®^ 100 mg/mL; 100 mg/kg; CP-Pharma Handelsgesellschaft mbH, Burgdorf, Germany), xylazine (Rompun^®^ 20 mg/kg; 2.8 mg/kg KGW; CP-Pharma Handelsgesellschaft mbH, Burgdorf, Germany), and Midazolam-ratiopharm^®^ (5 mg/5 ml; 0.7 mg/kg KGW; Ratiopharm GmbH, Ulm, Germany). Five minutes before initiation of anesthesia, animals received atropine (Atropinsulfat 0.5 mg/mL, 0.05 mg/kg KGW; B. Braun SE, Melsungen, Germany) and meloxicam (Metacam^®^, 2 mg/mL, 1 mg/kg KGW; Boehringer Ingelheim Vetmedica GmbH, Ingelheim am Rhein, Germany) subcutaneously. During the procedure and postoperatively, the animals were placed on a heated blanket (surface temperature 35°C), and their corneas were protected from drying out during anesthesia using eye ointment (Bepanthen^®^ Bayer, Leverkusen, Germany). For postoperative analgesia, the mice received daily meloxicam (Metacam^®^, 2 mg/mL, 1 mg/kg KGW) subcutaneously during the first 3 days after surgery. After ensuring the depth of anesthesia by checking the inter-toe reflex, the animals' abdomens were shaved and disinfected with braunol (B. Braun, Melsungen, Germany). The abdomen was opened along the linea alba, and after resection of all mLN, the transplant was inserted into the mesentery, the intestine was placed in the peritoneum, and the abdomen was closed. The animals were under constant observation, including control of breathing and reflexes, and were placed on a warming blanket until they were fully awakened from the anesthesia. The recovery time from the anesthesia was between 45 and 90 min.

#### Disease activity index (clinical score)

B6J mice (*n* = 43, no control mice) were weighted and monitored 2, 4, 6, 8, 24, and 48 h after surgery by clinical score and lMGS (prior to subcutaneous meloxicam (Metacam^®^, 2 mg/mL, 1 mg/kg KGW) injection) and on the days 1, 4, 7, and 8 after *C. rodentium* infection. Moreover, the DAI of B6-*Il4/Il10*^−/−^ mice was assessed daily for 1 week during 2% DSS treatment and two times a week until the end of the experiment. The scoring included the evaluation of the activity and purity of the eyes, fur, body openings, and body weight in six severities (Table 1). Mice reaching endpoint criteria between score 3 and score 4, leads to the euthanasia of the animals.

**Table 1:** Clinical scoring.

**Score**	**Definition**
1	Fast running and active Fur: smooth and shiny Eyes: wide open and round Body openings clean Normal social contact
2	Active with slightly increased pauses 5–10% weight loss Fur: smooth and shiny Eyes: wide open and round Body openings clean Normal social contact
3	Inactive mice 10–20% weight loss Fur: dull and ruffled Eyes: almond-shaped lid position Diarrhea Wound healing: swelling, redness
4	Inactive mice More than 20% body weight loss Fur: dull and ruffled Eyes: slit-like lid position Severe diarrhea

#### Live mouse grimace scale

The lMGS was taken at similar time points to the DAI assessment. It consists of orbital tightening, nose bulge, cheek bulge, ear position, and whisker change (20). The individual parameters were scored 0, 1, and 2 while the observed mouse was sitting separately in an extra empty cage for at least 30 s. The final lMGS score is calculated by the average of all five individual scores. The lMGS is divided into three degrees of severity. 0: all mentioned features are inconspicuous. 1: slight changes in the five features show moderate pain. 2: strong changes in the mice features show severe pain (20).

#### Chronic colitis induction

After 8 weeks post-operation, chronic colitis was induced by the application of 2% DSS (mol wt 36,000–50,000; MP Biomedicals, Eschwege, Germany) via drinking water on four consecutive days. Four weeks after DSS-induced colitis, B6-*Il4*/*Il*10^−/–^ mice were sacrificed.

#### Citrobacter rodentium infection

*C. rodentium* ICC180 (33) was kindly provided by M. Lochner. The bacteria were cultured in a lysogeny broth medium, and the mice were treated with 1 × 10^9^ Bacteria/mouse in 100 μl PBS intragastrically by oral gavage.

#### Histology

Colon samples were prepared as modified “swiss role”, fixed in neutral buffered 4% formalin, embedded in paraffin, sectioned at 5–6 μm, and stained with hematoxylin and eosin. Histology slides of chronic colitis mice were blindly scored for ulceration, hyperplasia, severity, and the involved area as described previously (11). Briefly, each parameter was graded from physiological (0) to severe changes (3) and added in a total score from 0 to 12. Colon sections were scored separately for the proximal, middle, and distal parts. A total colon score was calculated by adding all three colon sections with a maximum of 36 (Table 2).

**Table 2:** The chronical colitis histology scoring of DSS-model.

**Organ**	**Severity**	**Hyperplasia**	**Ulceration**	**Area**	**Total**
Proximal colon	0–3	0–3	0–3	0–3	36
Middle colon	0–3	0–3	0–3	0–3	
Distal colon	0–3	0–3	0–3	0–3	

Histology slides of *C. rod*.-infected animals were scored for epithelial hyperplasia (score marks the hyperplasia level above control: 0 = no change, 1 = 1–50%, 2 = 51–99%, 3 ≥ 100%) and cellular infiltration of mononuclear cells (0 = no infiltration, 1 = mild infiltration, 2 = moderate infiltration, 3 = severe infiltration). The scores are added up to a maximum of 6 (Table 3).

**Table 3:** The acute colitis histology scoring of *C. rod*. model.

**Organ**	**Hyperplasia**	**Cellular infiltration of mononuclear cells**	**Total**
Colon	0–3	0–3	6

#### Statistical analysis

Sample sizes were calculated according to a power analysis (power = 0.9 and α = 0.05) using G^*^Power Software (HHU Düsseldorf, Düsseldorf, Germany). All statistical analyses were performed using GraphPad Prism^®^8 software (GraphPad Software, Boston, MA, USA). Data were tested for normality with the Shapiro–Wilk normality test. Data were analyzed using the Kruskal–Wallis test together with Dunn's multiple comparisons test. Significance levels were set at 5%. Statistical differences are indicated by ^*^P < 0.05; ^**^P < 0.01; ^***^P < 0.001; ^****^P < 0.0001.

## Results

### Assessment of clinical parameter and live mouse grimace scale after intestinal surgery

**Figure 2:**
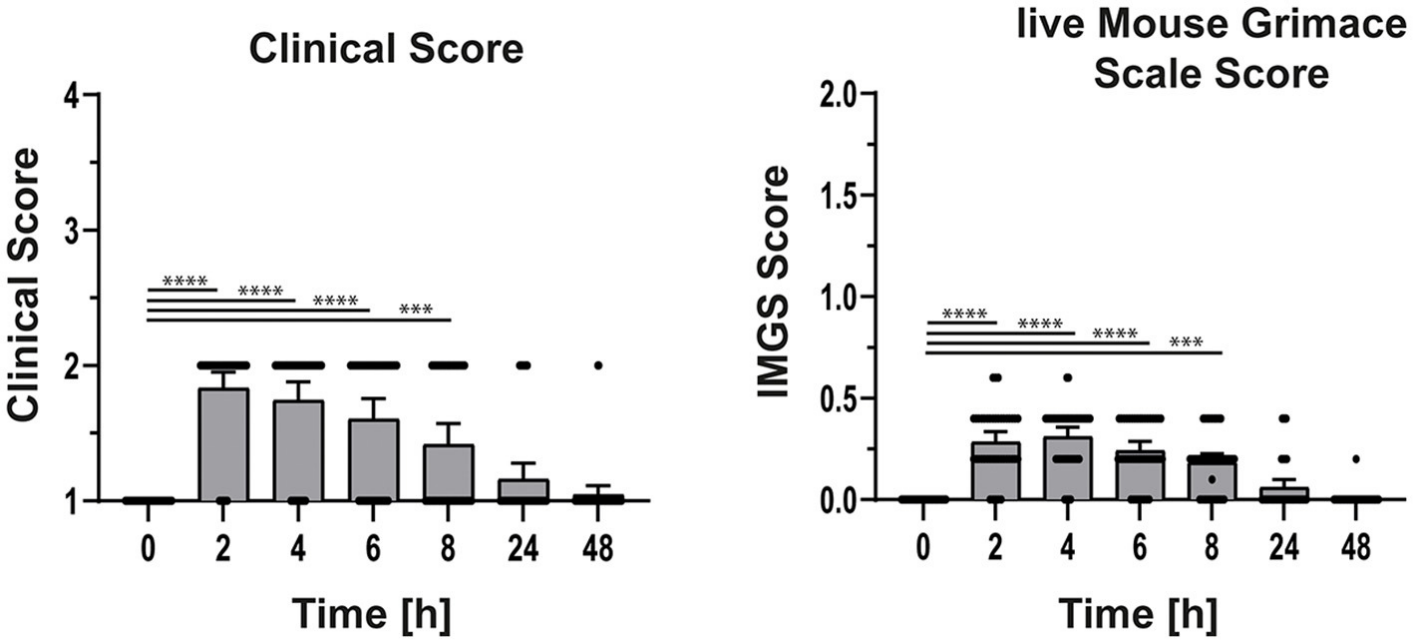
Clinical and lMGS score of the mice 48 h after a surgical intervention. Mice were scored before and 2, 4, 6, 8, 24, and 48 h after surgery. After scoring mice received meloxicam before surgery, 24 and 48 h after surgery. Data are shown as mean ± 95% confidential intervals (*n* = 43). Statistically significant differences are indicated by ***P < 0.001, ****P < 0.0001.

For welfare assessment in this study, the lMGS and clinical score were assessed before (bsl) and every 2, 4, 6, 8, 24, and 48 h after abdominal surgery. According to the clinical score sheet, nominal scores for weight loss, posture, and social behavior (Table 1) were summed. The highest clinical score of 1.8 as well as the highest lMGS of 0.3 was detected 2 h after surgery (Figure 2). Both scores decreased over a period of 8 h. The increase in clinical score is dependent on limited mobility and activity after surgery as well as fur care (2 h 35 animals out of 43; 4 h 32 out of 43; 6 h 26 out of 43; 8 h 18 out of 43, 24 h 7 out of 43, 48 h 2 out of 43). The lMGS showed within the first 6 to 8 h of alteration within the orbital tightening (33/43), nose bulge (41/43), cheek bulge (35/43), and ear position (13/43). Whisker change was not observed during the observation time. After 24 and 48 h, most of the animals showed no sign of pain or severity.

### Assessment of pain in DSS triggered chronic colitis

**Figure 3:**
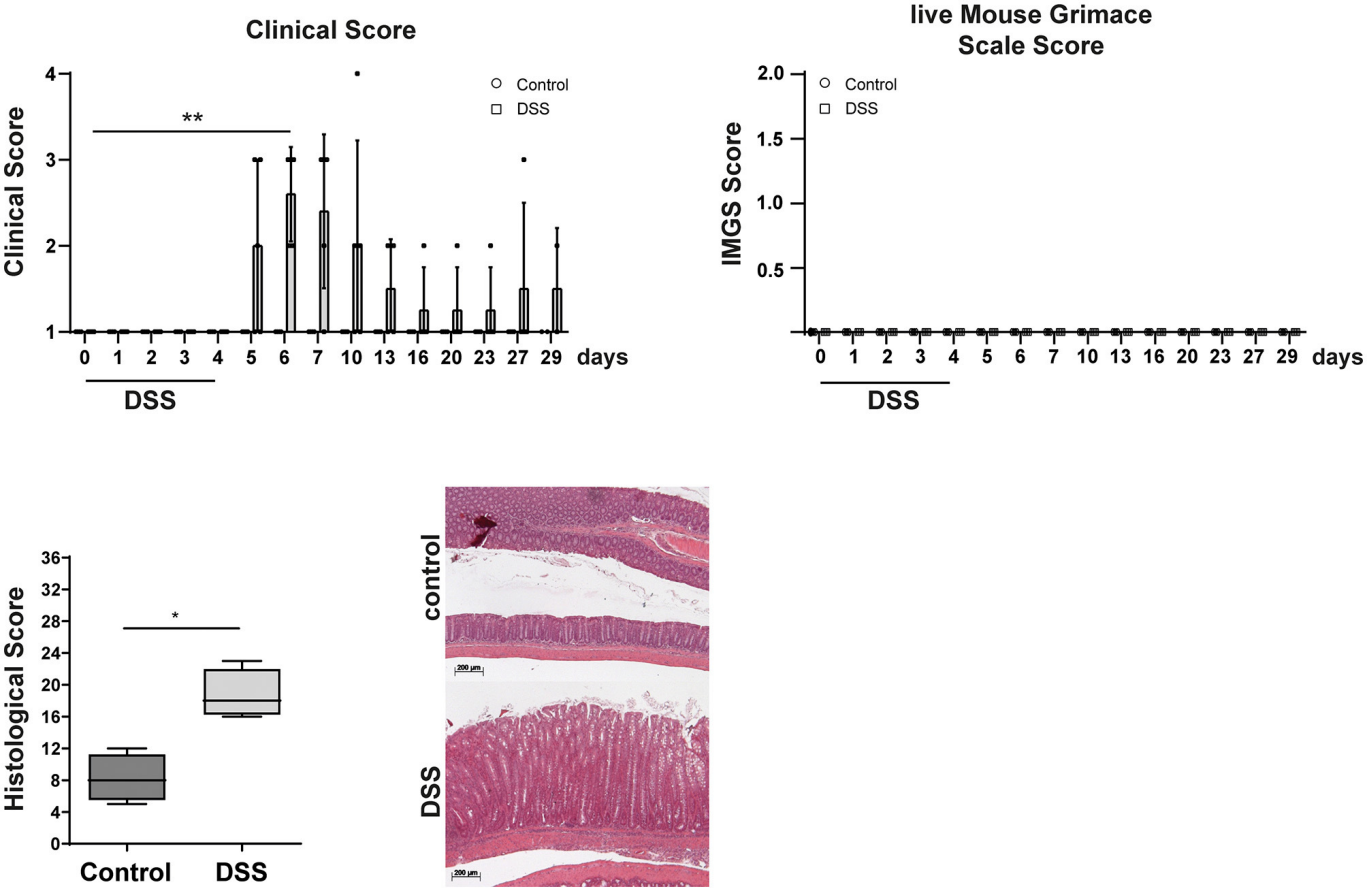
Clinical, lMGS, and histological scoring of B6-*Il4*/*Il*10^−/−^ mice during chronic colitis development. Mice were scored daily 7 days after starting DSS treatment. During the next weeks, the score was assessed at least twice a week. After 4 weeks, colon samples were taken and histological scoring was performed. Control mice received water. DSS-treated mice developed chronic colitis with the characteristic of strong epithelial hyperplasia in inflamed mice in contrast to the healthy mice. Clinical score is shown as mean ± SD (*n* = 4–5). lMGS data are shown as mean ± 95% confidential intervals (*n* = 4–5). Histological scores presented in the box and whiskers plots are the medians with minimum and maximum (*n* = 4). Representative images of hematoxylin and eosin-stained colon sections of transplanted B6-*Il4*/*Il*10^−/−^ mice. Statistically significant differences are indicated by *P < 0.05; **P < 0.005.

Eight weeks after surgery, B6-*Il*4/*Il*10^−/−^ mice were treated with DSS to trigger chronic intestinal colitis. As these mice developed intestinal inflammation first during the acute phase of DSS treatment (days 4–10) followed by chronic inflammation, clinical scores including weight loss, lMGS, and histological score were analyzed. The animals were weighed and scored every day during the first 7 days and afterward 2 times a week. Controls, which received water, neither showed changes in clinical scoring nor in lMGS scores (Figure 3). DSS treatment results in weight loss around day 5 and is one of the prominent characteristics of acute colitis induction (13). As the weight loss is included in the clinical score, increased score values were measured in all mice on days 6 and 7. Two mice showed increased scores until the end of the experiment, and one animal reached the endpoint criteria on day 8. No other parameter of the clinical score was altered during colitis induction. However, using the lMGS score, no scoring parameters changed during the acute phase as well as the chronic phase of the experimental time (Figure 3).

The histological scoring revealed an increased inflammation in colitogenic mice compared with control animals (Figure 3). The histopathological analysis revealed moderate score values in all colitis-induced mice. Lesions observed were characterized by lymphocytic infiltration, crypt hyperplasia, and goblet cell loss. Thus, the lMGS score seems to be not sensitive enough to detect pain during and after colitis induction, whereas the clinical scoring and, here, especially the weight loss indicate a decreased wellbeing.

### Valuation of severity after *Citrobacter rodentium* infection

**Figure 4:**
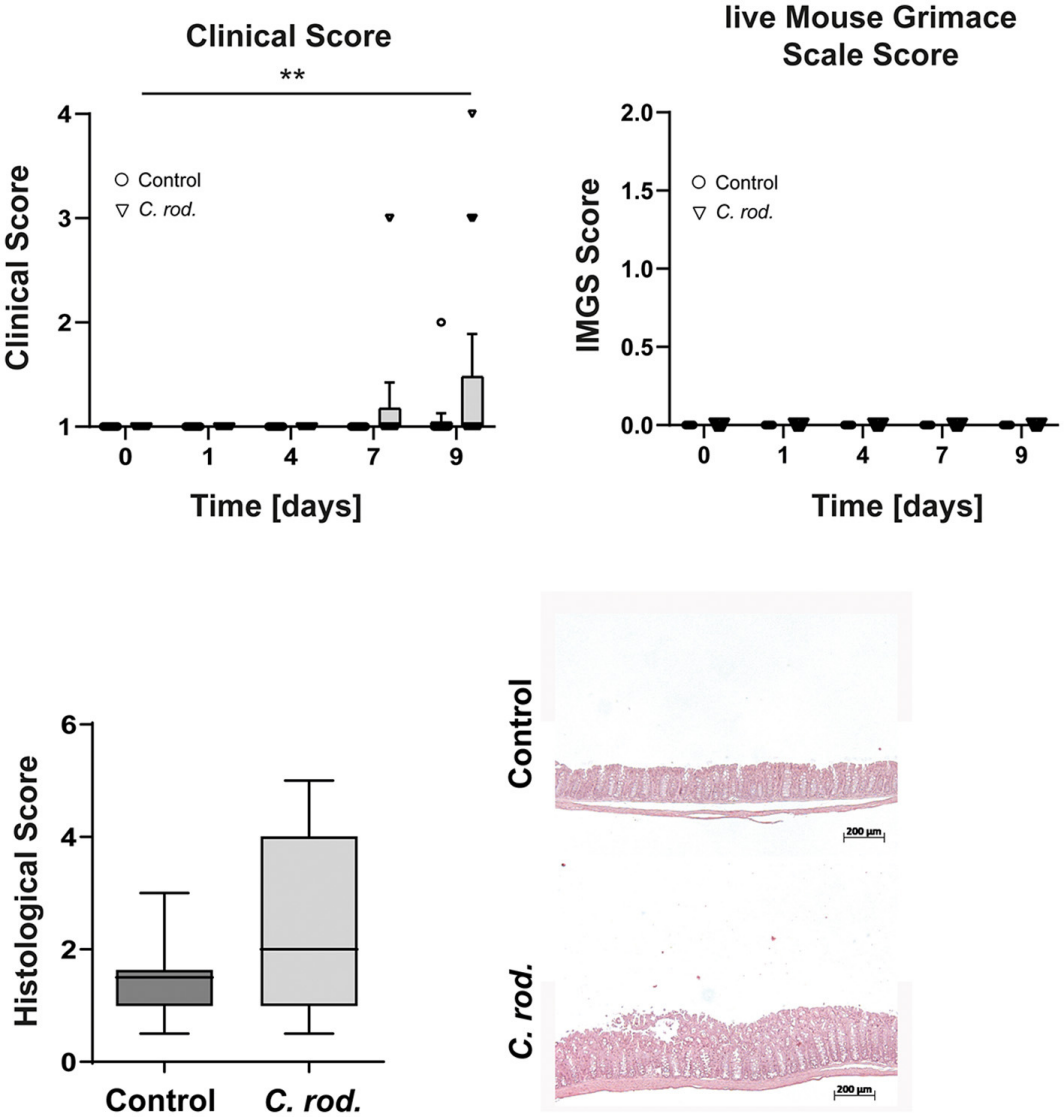
Clinical, lMGS, and histological scoring of B6J mice during acute inflammation after *C. rod*. infection. The scores were assessed at least five times in 10 days. Data were shown as mean ± 95% confidential intervals (*n* = 23–24). After 10 days, colon samples were taken and histological scoring was performed. The histological score demonstrates a moderate acute inflammation with the characteristic of immune cell infiltration and hyperplasia in *C. rod*. treated mice in contrast to the untreated mice. Histological scores presented in the box and whiskers plots are the medians with minimum and maximum (*n* = 9–10). Representative images of hematoxylin and eosin stained colon sections of *C. rod*. infected mice. Statistically significant differences are indicated by **P < 0.005.

B6J mice were infected orally with *C. rodentium* 4 weeks after surgery. *C. rodentium* is known to induce intestinal inflammation by activating the innate as well as the adaptive immune system (34). Therefore, the clinical scores including the weight loss, lMGS, and histological score were analyzed. The animals were weighed and scored 4 times after infection (Figure 4). Control animals, which received PBS, showed no clinical signs and remain stable in weight during the experiment (Figure 4). The clinical score increased in *C. rodentium*-infected mice at day 7 to a maximum score of 3 (*n* = 2 mice), whereas most animals showed a score of 1 (*n* = 21). The weight loss of ~15% was responsible for the increased clinical score. At day 9 after infection, an increased clinical score was detected in 5 mice (score 3–4) and 18 mice were scored 1. Anyway, no increased score values in the lMGS were measured (Figure 4). Infected mice revealed an increased histological score compared with control animals (Figure 4). The histopathological analysis revealed moderate inflammation in all *C. rodentium*-treated animals. Observed lesions were characterized by mononuclear cell infiltration and crypt hyperplasia. Thus, during *C. rod*. infection, the lMGS score was not sensitive to detect pain, whereas the clinical scoring, and, here, especially the weight loss, was an indicator of disease severity.

## Discussion

The welfare assessment of laboratory animals is essential for judging the health and condition of animals in experiments. For this, several methods have been established such as the lMGS, which can be used to identify acute pain, and suffering after, e.g., surgical interventions or other painful conditions. In this study, we examined pain and wellbeing in mice using the lMGS and clinical scoring after abdominal surgery and during DSS- and *C. rod*.-induced intestinal inflammation. Clinical scoring revealed impaired wellbeing after surgical intervention, during acute DSS colitis, and after *C. rod*. infection. In contrast, lMGS only detected pain within the first hours after surgical intervention but not during DSS-induced chronic colitis or intestinal *C. rod*. infection.

Over the last years, pain and severity assessment has come into focus. Several methods such as burrowing, nesting, or the MGS were used to elucidate their possible usage in pain assessment in mice (21). The MGS has been applied in different mouse models to detect pain after vasectomy (35), thoracotomy (36) during the development of neuropathic pain, in multiple sclerosis, or in a model for sickle cell disease (37–39). In addition, various biological and environmental factors, such as strain, sex, or the presence of the observer, were elucidated (40, 41). Baseline MGS scores between C57BL/6 mice, CD1, and C3H/He animals were compared as well as sex differences in these strains (42). Differences were detected between the strains and between the sex of BALB/c, CD1, and C3H/He mice, whereas no differences between male and female mice were observed in C57BL/6 animals (42, 43). In our study, different B6 background strains were used for different experiments; however, no differences were detectable between male and female mice during DSS-triggered intestinal inflammation. Furthermore, in our hands the scoring was observer independent as only one observer scored the mice. However, a previous study found a reduction of scores in the presence of a male observer (44). Since Langford et al. developed a retrospective MGS, grimace score systems, e.g., rats, ferrets, sheep, and horses were established (22–25). Using this technique, researchers can detect pain but also distress which enables them to refine pain management and improve animals' welfare. However, most of the studies which performed MGS scoring used pictures or video recording (41), and the lMGS was developed for real-time cage-side analysis (28, 31). In rats, differences between the control and analgesic–treated groups using cage-side interval observations were detectable, but cage-side point observations showed substantial variability (31). In all studies, so far, the lMGS showed lower score values compared to the MGS, but the advantage for the scientist might be an earlier pain detection, enabling an immediate intervention (28, 42, 45).

In the chronic colitis model of this study, in which mice received DSS for colitis induction, the clinical score detected impaired animal welfare, whereas the lMGS score indicated no pain. This might be due to the fact that the mice did not experience pain or that the lMGS is not sensitive to pain detection in this model. In another study investigating the best disease indicator during colorectal cancer development in mice, the authors reported a good disease severity correlation between a model-specific disease activity index and the outcomes of colonoscopy and tumor development. While the retrospective MGS revealed signs of pain alleviation in animals treated with buprenorphine, the lMGS did not (28). Furthermore, in a study investigating pain due to repeated intraperitoneal injections in mice, the authors showed elevated MGS in mice receiving CCl_4_, which is a model for liver fibrosis, but not in the control animals receiving the vehicle substance (oil only) (46). Very recently, Vezza et al. showed increased facial pain expressions on day 4 after DSS treatment in a video-based retrospective analysis (47). This, in line with our results, leads to the suggestion that the lMGS is not sensitive enough for the detection of intestinal inflammatory pain and the retrospective MGS seems to be a better indicator.

In the part of the study where we monitored pain after bacterial *C. rod*. infection, the clinical score increased but the lMGS did not detect signs of pain. However, in studies investigating pain in other infection models, the MGS detected pain during disease progression in a sepsis model (48, 49) or after intracranial LCMV infection (50), but these mice models are severe animal models, related to severe pain and suffering, and the endpoint is the death of the animals.

As the MGS was developed to detect postoperative pain (20), we were able to detect pain directly after abdominal surgery using the lMGS. These slightly elevated scores could be due to anesthesia, as elevated MGS values have been detected after ketamine/xylazine or isoflurane anesthesia (51, 52) or to the inefficiency of meloxicam in this model (53, 54). But increased concentrations of meloxicam result in toxicity after subcutaneous administration (55). Analgesics such as opioids are suggested to have a sedative effect resulting in increased scores in a rat tumor model (56). Other analgesics such as buprenorphine seem to be more effective in reducing the MGS score (53, 54, 57). However, for an improved pain detection and welfare assessment in mouse models with inflammatory intestinal pain, other behavioral methods are available. Spontaneous, species-specific behaviors such as burrowing and nesting are well-reported indicators of postoperative pain. In a study, investigating burrowing behavior during DSS colitis, the authors reported a delayed onset of burrowing behavior in mice, indicating that the animals experienced pain (58). Recently, in our laboratory, we investigated the nesting behavior of mice during the course of DSS colitis. Here, we showed increased time intervals that the mice needed to integrate items into their nests in the home cage. In another study from our laboratories, we showed a DSS dose-dependent reduction of wheel-running behavior in mice suffering from acute DSS colitis. In addition, wheel-running behavior is a well-described parameter for the detection of pain, as recently reviewed here (59).

### Limitations and perspectives

A limitation of this study is that only a cage-side live score was assessed. Additional retrospective MGS detection would have increased the validity of the results. Furthermore, analysis of different analgesia regimes during intestinal inflammation will be helpful for a better refinement protocol in mice (60).

In summary, the results of our study showed, that the lMGS is a suitable tool for the detection of postoperative pain but not for intestinal inflammatory pain. Further studies are required to analyze whether the retrospective MGS, based on video recordings, is more sensitive for pain detection.

## Data availability statement

The original contributions presented in the study are included in the article/supplementary material, further inquiries can be directed to the corresponding author.

## Ethics statement

This study was conducted in accordance with German animal protection law and with the European Directive 2010/63/EU. All experiments were approved by the Local Institutional Animal Care and Research Advisory committee (Hannover Medical School) and permitted by the Lower Saxony State Office for Consumer Protection and Food Safety (LAVES; file number: 20/3445).

## Author contributions

CH and MB conceived and designed the experiments and wrote the manuscript. PP, JG, and EZ performed the experiments and analyzed the data. MB, CH, and AB supervised the work. All authors contributed to the article and approved the submitted version.
